# Supplementary figures and images for: Evaluation of high-resolution melt curve analysis for rapid differentiation of Campylobacter hepaticus from other species in birds
Source: PLoS One. 2021 May 13;16(5):e0251328. doi: 10.1371/journal.pone.0251328 (PMC8118346; doi:10.1371/journal.pone.0251328)

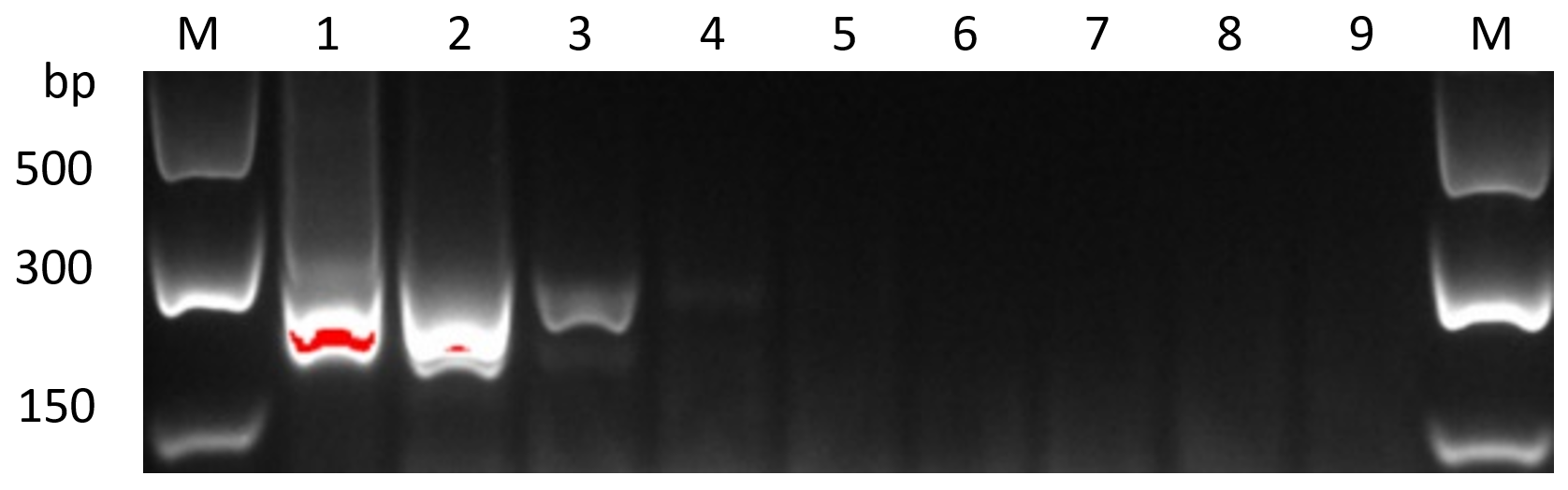

Supplement: S1 Fig — Lane M: DNA ladder, Lane 1–9: C. hepaticus DNA at 1 ng, 10−1 ng, 10−2 ng, 10−3 ng, 10−4 ng, 10−5 ng, 10−6 ng, 10−7 ng and 10−8 ng concentrations. (TIF) [file pone.0251328.s001.tif]
