# Supplementary material for: Evaluation of high-resolution melt curve analysis for rapid differentiation of Campylobacter hepaticus from other species in birds
Source: PLoS One. 2021 May 13;16(5):e0251328. doi: 10.1371/journal.pone.0251328 (PMC8118346; doi:10.1371/journal.pone.0251328)
Supplement: Attachment — Submitted filename: Response to reviewers Comments.docx [file pone.0251328.s002.docx]

**Response to reviewer’s comments**

Responses to the journal requirements and reviewer’s comments in a point-by-point manner is provided in red font.

Journal Requirements:

When submitting your revision, we need you to address these additional requirements.

1. Please ensure that your manuscript meets PLOS ONE's style requirements, including those for file naming. The PLOS ONE style templates can be found at

https://journals.plos.org/plosone/s/file?id=wjVg/PLOSOne_formatting_sample_main_body.pdf and

[https://journals.plos.org/plosone/s/file?id=ba62/PLOSOne_formatting_sample_title_authors_affiliations.pdf](about:blank)

 The manuscript was re-formatted based on PLOS ONE’s style.

2. In your Methods section, please provide additional details regarding the chicken and wild birds, including the number and species, used in your study and ensure you have described the source. For more information regarding PLOS' policy on materials sharing and reporting, see https://journals.plos.org/plosone/s/materials-and-software-sharing#loc-sharing-materials.

Required information including the number of tested chickens, wild birds, species and location of collected samples were added to the text lines 87-90.

3. In your Methods section, please provide additional information regarding the permits you obtained for the work. Please ensure you have included the full name of the authority that approved the field site access and, if no permits were required, a brief statement explaining why.

  The institutional permit for sample collection was added in the text lines 84-86.

4. PLOS ONE now requires that authors provide the original uncropped and unadjusted images underlying all blot or gel results reported in a submission’s figures or Supporting Information files. This policy and the journal’s other requirements for blot/gel reporting and figure preparation are described in detail at https://journals.plos.org/plosone/s/figures#loc-blot-and-gel-reporting-requirements and https://journals.plos.org/plosone/s/figures#loc-preparing-figures-from-image-files. When you submit your revised manuscript, please ensure that your figures adhere fully to these guidelines and provide the original underlying images for all blot or gel data reported in your submission. See the following link for instructions on providing the original image data: <https://journals.plos.org/plosone/s/figures#loc-original-images-for-blots-and-gels>.

Gel image was provided as supporting information (S1 Fig) and the rest of figures were re-numbered accordingly.

In your cover letter, please note whether your blot/gel image data are in Supporting Information or posted at a public data repository, provide the repository URL if relevant, and provide specific details as to which raw blot/gel images, if any, are not available. Email us at plosone@plos.org if you have any questions.

Done.

 Your ethics statement should only appear in the Methods section of your manuscript. If your ethics statement is written in any section besides the Methods, please move it to the Methods section and delete it from any other section. Please ensure that your ethics statement is included in your manuscript, as the ethics statement entered into the online submission form will not be published alongside your manuscript.

 Done.

6. We note that you have included the phrase “data not shown” in your manuscript. Unfortunately, this does not meet our data sharing requirements. PLOS does not permit references to inaccessible data. We require that authors provide all relevant data within the paper, Supporting Information files, or in an acceptable, public repository. Please add a citation to support this phrase or upload the data that corresponds with these findings to a stable repository (such as Figshare or Dryad) and provide and URLs, DOIs, or accession numbers that may be used to access these data. Or, if the data are not a core part of the research being presented in your study, we ask that you remove the phrase that refers to these data.

  The related phrase was removed from the text (line 179-180).

5. Review Comments to the Author

Reviewer #1: PONE-D-21-00269: Evaluation of high-resolution melt curve analysis for rapid differentiation of Campylobacter hepaticus from other species in birds

The study evaluated high-resolution melt curve analysis to differentiate Campylobacter hepaticus from Campylobacter coli and Campylobacter jejuni. However, the authors have not conducted any other method comparison study to call the overall method “rapid.” Therefore, this rapid differentiation can be changed or removed or else should add about facts on time duration and justify accordingly.

To address this comment, additional text has been added to the discussion lines 245-249 and 253-256 and provided references that compared the time required to complete PCR-HRM with PCR gel electrophoresis (Thomsen et al., 2012 and Sarker et al., 2014) that confirms PCR-HRM is rapid when compared with PCR gel electrophoresis.

The study does not include any experiment to find out the specificity of the assay. Also, the inclusion of other Campylobacter species and different related genus is important to assess the possibility of false-positive results.

To evaluate the specificity of the assay, DNA was extracted from seven bacterial strains of genetically similar genera (*Klebsiella*, *Pseudomonas*, *Enterobacter*, *Staphylococcus*, *streptococci*, *E. coli* and *Pasteurella*) and tested in PCR-HRM to address this comment. This is now added to the text in lines 154-157 and 215-218 of new version of manuscript.

In conclusion, “….rapid and sensitive diagnostic tool….” This can be rephrased or possibly soften appropriately

This comment is addressed above (first comment).

Change the figure no. to maintain the order

One figure (gel image- Fig 4) was provided as supporting information file and the rest of figures were re-numbered accordingly to maintain the order in the text.

Lines 204-205: Rephrase this sentence

To address this comment, the sentence was revised in lines 211-212 of new version of manuscript.
